# Kinobead Profiling Reveals Reprogramming of BCR Signaling in Response to Therapy within Primary CLL Cells

**DOI:** 10.1158/1078-0432.CCR-21-0161

**Published:** 2021-08-11

**Authors:** Adam J. Linley, Laura I. Karydis, Anil K. Mondru, Annalisa D'Avola, Humood Al Shmrany, Silvia Cicconi, Rebecca Griffin, Francesco Forconi, Andrew R. Pettitt, Nagesh Kalakonda, Andrew C. Rawstron, Peter Hillmen, Andrew J. Steele, David J. MacEwan, Graham Packham, Ian A. Prior, Joseph R. Slupsky

**Affiliations:** 1 Department of Molecular Physiology and Cell Signaling, Institute of Systems, Molecular and Integrative Biology, University of Liverpool, Liverpool, United Kingdom.; 2 School of Cancer Sciences, Cancer Research UK Centre, University of Southampton, Southampton, United Kingdom.; 3 Department of Molecular and Clinical Cancer Medicine, Institute of Systems, Molecular and Integrative Biology, University of Liverpool, Liverpool, United Kingdom.; 4 Department of Medical Laboratory Sciences, College of Applied Medical Sciences, Prince Sattam Bin Abdulaziz University, Al-Kharj, Saudi Arabia.; 5 Cancer Research Clinical Trials Unit, University of Liverpool, Liverpool, United Kingdom.; 6 Department of Haematology, Leeds Teaching Hospitals NHS Trust, Leeds, United Kingdom.; 7 Faculty of Medicine and Health, School of Medicine, University of Leeds, Wellcome Trust Brenner Building, Leeds, United Kingdom.; 8 Department of Pharmacology and Therapeutics, Institute of Systems, Molecular and Integrative Biology, University of Liverpool, Liverpool, United Kingdom.

## Abstract

**Purpose:**

B-cell receptor (BCR) signaling is critical for the pathogenesis of chronic lymphocytic leukemia (CLL), promoting both malignant cell survival and disease progression. Although vital, understanding of the wider signaling network associated with malignant BCR stimulation is poor. This is relevant with respect to potential changes in response to therapy, particularly involving kinase inhibitors. In the current study, we describe a novel high-resolution approach to investigate BCR signaling in primary CLL cells and track the influence of therapy on signaling response.

**Experimental Design:**

A kinobead/mass spectrometry–based protocol was used to study BCR signaling in primary CLL cells. Longitudinal analysis of samples donated by clinical trial patients was used to investigate the impact of chemoimmunotherapy and ibrutinib on signaling following surface IgM engagement. Complementary Nanostring and immunoblotting analysis was used to verify our findings.

**Results:**

Our protocol isolated a unique, patient-specific signature of over 30 kinases from BCR-stimulated CLL cells. This signature was associated with 13 distinct Kyoto Encyclopedia of Genes and Genomes pathways and showed significant change in cells from treatment-naïve patients compared with those from patients who had previously undergone therapy. This change was validated by longitudinal analysis of clinical trials samples where BCR-induced kinome responses in CLL cells altered between baseline and disease progression in patients failing chemoimmunotherapy and between baseline and treatment in patients taking ibrutinib.

**Conclusions:**

These data comprise the first comprehensive proteomic investigation of the BCR signaling response within CLL cells and reveal unique evidence that these cells undergo adaptive reprogramming of this signaling in response to therapy.

**Translational Relevance**

B-cell receptor signaling (BCR) is a pathogenetic driver of chronic lymphocytic leukemia (CLL) but has not been studied in the context of therapy resistance because of a lack of suitable proteome-based approaches. Here we describe mass-spectrometry identification of kinobead-isolated kinases as a technique to investigate signaling in CLL cells that have been stimulated by BCR engagement. We show the signature of isolated kinases in CLL cells from treatment-naïve patients is significantly different from that in cells from patients in receipt of therapy. Further longitudinal analysis of patient samples donated from clinical trials indicate that this difference results from chemoimmunotherapy and ibrutinib treatment, suggesting that BCR signaling undergoes a process of active rewiring in response to therapy. We propose these findings act as a basis to investigate effects of therapy on signaling in CLL cells.

## Introduction

The accumulation and survival of chronic lymphocytic leukemia (CLL) cells is strikingly dependent on signaling from activated cell surface receptors (1, 2). This applies particularly to the B-cell receptor (BCR) where antigen-dependent and antigen-independent activity has been linked to disease development (1, 3–5). For example, retained anti-IgM signaling capacity is associated with progressive disease (6), and kinase inhibitors (KI) targeted against BCR signaling pathways, especially the Bruton tyrosine kinase (BTK) inhibitor ibrutinib, can induce impressive clinical responses in patients with CLL and some subtypes of non–Hodgkin lymphoma (7–9). In addition to disease pathogenesis, altered signaling influences response to therapy. This is clearest in the context of acquired resistance to ibrutinib which is commonly associated with mutations within BTK itself or its downstream effector, phospholipase Cγ2 (PLCγ2), that result in production of proteins which retain activity but bind ibrutinib less avidly, or are hyperresponsive to upstream activating pathways, respectively (10–12).

Kinase activation and inhibition in primary CLL cells has generally been analyzed by examining kinase-substrate phosphorylation using phospho-specific antibodies to identify modified amino acids. While such an approach can provide important insight, interpretation of results can be challenging. For example, the activity of many kinases is influenced by multiple phosphorylation events which can be activating or inhibiting. Where phosphorylation at multiple sites on an individual kinase is detected, it can be difficult to determine whether these are on the same molecule, or whether subpopulations of kinases with distinct patterns of phosphorylation (and hence activity) co-exist. Furthermore, upstream kinases responsible for this phosphorylation can be inferred on the basis of substrate specificity, but redundancy among kinases (where several kinases may be able to modify an individual phospho-acceptor site) limits the utility of this approach. Finally, changes in phosphorylation will reflect changes not only in kinase activity, but also their counteracting phosphatases.

Kinobeads provide a powerful tool to probe kinase function (13–16). In this approach, cell lysates are incubated with beads coated with broad-specificity type 1 KI, allowing binding of a large proportion of the expressed kinome. Bound kinases can then be profiled using mass spectrometry (MS). Kinobead-MS technology was initially deployed to determine the profile of kinases affected by KI treatment of cells, including malignant hematologic cells, since binding of KI to target kinases prevents their subsequent capture by the kinobeads (17). However, these early studies did not address how KIs affect overall signaling in treated cells. Furthermore, kinase capture can also be influenced by changes in the abundance of the kinases and, in particular, active site conformation. Many kinases exist in an autoinhibited form and their activation requires conformational change. For example, BTK adopts multiple conformations allowing graded activation following cell stimulation (18), and ERK activation requires a MEK-dependent switch to an active conformation (19). Taking these considerations into account, subsequent studies have shown that kinobead technology can be used to probe kinase activation in response to stimulation of breast cancer cells and, in particular, to reveal adaptive “rewiring” of kinase networks following exposure to KI (13, 20).

Here, we have developed the kinobead approach for analysis of malignant B cells and used it for the first time to characterize signaling in primary CLL cells. We show kinobeads can be used to assess active site occupancy by KIs, including in ibrutinib-treated patients.

## Materials and Methods

### Cells

Primary cells from patients diagnosed with CLL according to iwCLL-2008 criteria were obtained from patients attending clinics at Royal Liverpool and Broadgreen Hospitals at the University of Southampton Hospital trust in the Mature Lymphoid Malignancies Observational Study (NIHR/UKCRN Portfolio ID: 31076). All patients provided informed consent. Samples were provided as heparinized whole blood from which peripheral blood mononuclear cells were isolated and cryopreserved as described previously (21). Alternatively, primary cells from patients recruited to the ARCTIC/ADMIRE (ISRCTN16544962) and IcICLLe (ISRCTN12695354) clinical trials were obtained from the UK CLL Clinical Trials BioBank. In all cases, cells were recovered and diluted as outlined before (21). The malignant B-cell lines MEC-1, JeKo-1, and MAVER-1 were acquired from the DSMZ (Leibniz, Germany) and cultured in RPMI1640(+Glutamax) medium supplemented with 10% FCF and 1% Pen/Strep (all Thermo Fisher Scientific). Cell line identity was confirmed using short tandem repeat analysis (Powerplex 16 System, Promega) and the absence of *Mycoplasma* was confirmed using Mycoplasma PCR detection kit (Applied Biological Materials).

### Characterization of primary CLL cells

Cells from non-trial patients underwent cell surface phenotyping by flow cytometry using anti-CD19, anti-CD5 (both BioLegend), anti-IgM (Dako), and appropriate isotype control antibodies. Signaling capacity was determined by measuring the proportion of malignant cells that were able to flux intracellular calcium (iCa^2+^) following treatment with F(ab')_2_ anti-IgM, as described previously (29) using a FACScan or FACS Calibur instrument (BD Biosciences). All flow cytometry data were analyzed using FlowJo (TreeStar).

### Treatments

Primary CLL samples, along with JeKo-1 and MAVER-1 cells, were treated with 20 μg/mL anti-IgM F(ab')_2_ or control F(ab')_2_ (both Southern Biotech, Cambridge Biosciences) for 5 minutes at 37°C/5% CO_2_. Experiments involving the treatment of CLL cells with IL4 were performed in a manner similar to before (22). Briefly, following recovery cells were treated with 10 ng/mL recombinant IL4 (R&D Systems) for 24 hours at 37°C/5% CO_2_ or left untreated. The following day cells were stimulation using 20 μg/mL anti-IgM F(ab')_2_ or control F(ab')_2_ for 5 minutes at 37°C/5% CO_2_ prior to lysis. For comparative purposes using immunoblotting, cells with treated with IL4 alone. MEC-1 cells were pretreated and incubated at 37°C/5% CO_2_ for 1 hour with 500 nmol/L ibrutinib or dasatinib prior to protein lysis.

### Protein lysis for kinobead analysis

Cells were washed twice using ice-cold PBS and resuspended in kinobead lysis buffer: 50 mmol/L HEPES (pH 7.5), 150 mmol/L NaCl, 0.5% [volume for volume (v/v)] Triton X100, 1 mmol/L EDTA, 1 mmol/L EGTA, 10 mmol/L NaF, 2.5 mmol/L NaVO_4_, 1× Protease Inhibitor Cocktail (Roche), and 1× Phosphatase Inhibitor Cocktails 2 and 3 (Sigma). Suspensions were incubated on ice for 10 minutes with regular mixing before sonication and clarification by centrifugation at 13,000 rpm for 10 minutes. The supernatant was collected and protein content quantified using BCA assay (Thermo Fisher Scientific). Lysates were stored at −80°C prior to analysis.

### Kinobead preparation

We employed a protocol similar to that described previously (13). The broad-spectrum KI Ki-NET (CTx-0294885; SYN-Kinase) was conjugated to NHS-activated Sepharose-4 Fastflow resin (GE Healthcare). Briefly, Ki-NET was solubilized in coupling buffer consisting of 50% dimethylformamide/50% sodium phosphate (pH 7.0). Sepharose was resuspended and transferred onto the filter of a 0.22 μmol/L pore filter flask (Corning). The filter was then washed twice with 0.5 mol/L NaCl, once with Millipore water and once using coupling buffer. The washed beads were recovered from the filter and added to the dissolved Ki-NET. To promote conjugation, 1 mol/L *N*(3Dimethylaminopropyl)-N'-ethylcarbodiimide hydrochloride (EDC) was added and Ki-NET beads were incubated overnight at room temperature in the dark with continuous end-on-end mixing. The following day, beads were washed twice and resuspended in coupling buffer containing 1 mol/L ethanolamine. EDC (1 mol/L) was added and the beads were incubated overnight as described above. Conjugated kinobeads were washed three times with coupling buffer, twice with Millipore water and once with 20% (v/v) ethanol before final resuspension in 20% ethanol and being stored at 4°C. For other kinobeads tested, the above process was used for the inhibitor VI16832 and purvalanol-B which were conjugated to EAH Sepharose, while bisindolylmaleimide-X (Bis-X), CZC8004, and pp58 were conjugated to a 1:1 mixture of NHS-activated Sepharose-4 Fastflow resin and EAH Sepharose.

### Isolation of kinases using kinobeads

Protein lysates were thawed on ice and the specific quantity required (3 mg for MEC-1 experiments, 1 mg for primary CLL) was aliquoted, diluted with kinome lysis buffer and adjusted to 1 mol/L NaCl. Polyprep columns (Bio-Rad) were placed in a rack and kinobeads were resuspended, applied carefully to the bottom of the column, and washed using a high salt buffer: 50 mmol/L HEPES (pH 7.0), 1 mol/L NaCl, 0.5% (v/v) Triton X-100, 1 mmol/L EDTA, 1 mmol/L EGTA. In a separate column, unconjugated Sepharose was added to create a block condition and washed using high salt buffer. Block columns were placed above the kinobead columns and lysates were carefully loaded. Once lysates had passed through both the block and kinobead columns, the block columns were discarded and the kinobead columns were washed using high salt buffer, then low salt buffer [50 mmol/L HEPES (pH 7.0), 150 mmol/L NaCl, 0.5% (v/v) Triton X-100, 1 mmol/L EDTA, 1 mmol/L EGTA] and then SDS wash buffer [low salt buffer supplemented with 0.1% (weight/volume; w/v) SDS]. Columns were stoppered to prevent flow-through before the addition of elution buffer [0.5% (w/v) SDS, 1% (v/v) β-mercaptoethanol, 0.1 mol/L Tris-HCl (pH 6.8) in LC/MS grade water]. Columns were capped and incubated at 98°C for 15 minutes before being collected into low-bind Eppendorf tubes.

### Processing of kinobead elutions

Elutions from kinobeads were initially reduced by adding 5 mmol/L DTT and incubating at 60°C for 25 minutes. Samples were alkylated with 20 mmol/L iodoacetamide and incubated at room temperature for 30 minutes in darkness. Alkylation was inhibited by adding another 5 mmol/L DTT and incubation at room temperature for 5 minutes in darkness. Samples were loaded into concentrator spin-columns (Millipore) and centrifuged at 3,000 rpm for 30 minutes at 4°C. Concentrated samples were collected into low-bind Eppendorf tubes. To precipitate proteins, samples underwent initial spin-washes using methanol, chloroform, and water (all LC/MS grade) before centrifugation at 13,000 rpm for 10 minutes at 4°C. The interphase layer (protein) was collected and washed using MS-grade methanol. Samples were dried by speed vacuum before resuspension in 50 mmol/L HEPES (pH 8.0) and the addition of 500 ng Trypsin Gold (Promega). Pellets were incubated overnight at 37°C. The following day, peptide samples were washed three times using water-saturated ethyl acetate to remove Triton X-100 before drying with a speed vacuum. Peptides underwent desalting using C-18 spin columns (Thermo Fisher Scientific). Columns were equilibrated using a buffer of 5% acetonitrile/0.5% trifluoroacetic acid. Sample pellets were resuspended in this buffer and loaded into prepared spin columns and centrifugation at 4,400 rpm for 1 minute. Columns underwent further washes using equilibration buffer before undergoing elution using 50% acetonitrile and stored at 80°C prior to MS.

### MS sample testing and data analysis

Peptide samples were supplied to the WPH Proteomics Facility of the University of Warwick where they underwent final preparation and MS analysis using an Orbitrap Fusion instrument (Thermo Fisher Scientific) as outlined previously (23). Data files generated by MS (RAW) were analyzed using MaxQuant software (v1.5.3.8; Max Planck Institute). Peptide intensities involving serine, threonine, tyrosine phosphorylation alongside oxidation and acetylation were used as variable modifications, while carbamidomethylation to cysteine was using as a fixed modification. Protein identification was made by comparison with a Uniprot human proteome database. A 1% FDR was used for all searches. Changes in kinase levels were determined by creating ratios by comparing sIgM-stimulated CLL cells with the relevant control antibody-treated cells for each patient. Directionality of response was achieved by converting ratios to log_2_ scale. These data were then used to create heatmaps using Multi-experiment Viewer (MeV) software (tm4.org).

### Immunoblotting

For direct analysis, cells were lysed using RIPA buffer whereas for kinobead analysis, elutions from kinobeads were mixed with an equal volume of 2× loading buffer prior to SDS-PAGE. Immunoblotting was performed using anti-phospho-SYK (Y525/6), anti-SYK, anti-phospho-BTK (Y223), anti-BTK, anti-phospho-GAB1 (Y627), anti-GAB1, anti-phospho-TBK1, anti-TBK1, anti-phospho-PKAC, anti-PKACα, anti-phospho-ERK1/2 (Y202/T204), anti-ERK, anti-phospho-AKT (S473), and anti-AKT, (all Cell Signaling Technologies). Anti-actin (Sigma) and anti-HSC70 (Insight Biotechnology) were used as loading controls. Bound primary antibodies were detected using species specific fluorophore-conjugated secondary antibodies (LI-COR Biosciences) and imaged using a LI-COR Odyssey CLx instrument.

### Nanostring kinase mRNA analysis

RNA was extracted using an RNA-Easy kit (Qiagen) in accordance with the manufacturer's protocol. RNA quantity and quality were assessed using a Nanodrop 1000 instrument (Thermo Fisher Scientific). Kinase gene analysis was carried out using nCounter XT HuV2 Kinase arrays measuring mRNA abundance of the 535 possible kinases and pseudokinases expressed by the human genome (Nanostring) according to the manufacturer's protocol. A total of 100 ng of RNA was ligated to capture and reporter probes by incubation at 65°C for 22 hours using a Veriti Thermal Cycler (Thermo Fisher Scientific). Preparation of array cartridges was performed using a Nanostring PrepStation and read using an nCounter reader. Data were analyzed using nSolver software (v3.0; Nanostring).

### Phosphatase analysis

For surface expression analysis, cells were prepared as outlined above and 1 × 10^6^ were aliquoted into FACS tubes. Cells were pelleted by centrifugation and each tube was resuspended in flow buffer [1% (w/v) BSA, 4 mmol/L EDTA, and 0.15 mmol/L NaN_3_ in PBS] containing anti-CD19 and anti-CD5 antibodies for CLL cell detection. Antibodies against specific key markers and surface phosphatases were then used in various combinations. Cells were incubated on ice in the dark for 15 minutes prior to washing with flow buffer. Cells were resuspended in flow buffer and analyzed using a Canto cytometer. Data were analyzed using FlowJo.

For intracellular phosphatases, lysates were analyzed by immunoblotting with the following antibodies: anti-phospho-SHIP1, anti-phospho-SHP1, anti-phospho-LYN (Y507; all Cell Signaling Technology), anti-SHP1, anti-phospho-LYN (Y396; both Abcam), anti-SHIP1, anti-LYN (both Insight Biotechnology), anti-phospho-PTPN22 (R&D Systems), and anti-GAPDH (Thermo Fisher Scientific). Primary antibodies were probed using species-specific HRP-conjugated secondary antibodies (Dako) and viewed using a Chemidoc imager (Bio-Rad).

### Data availability

Kinome signatures detected by kinobead-MS and Nanostring are listed in Supplementary Data S1–S3. The MS proteomics data (.RAW files) have been deposited to the Proteome Xchange Consortium via the PRIDE partner repository with the dataset identifier PXD027131. For original data, please contact alinley@liverpool.ac.uk.

## Results

### Validation of kinobeads for the analysis of malignant B cells

**Figure 1.**
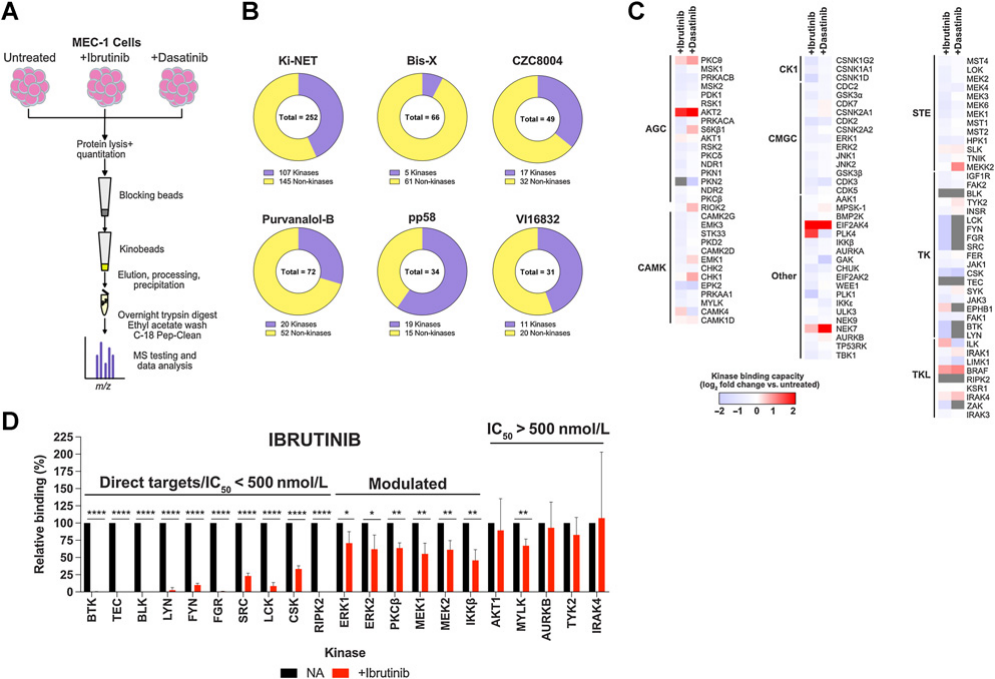
Kinobead analysis of MEC-1 cells. **A,** Schematic of the kinobead-MS approach for MEC-1 cells. **B,** Pie graphs illustrating the numbers of kinase and non-kinase proteins isolated from MEC-1 cell lysates using six different kinobeads. **C,** Heatmaps constructed using average data from experiments involving MEC-1 cells (*n* = 3), which were treated with 500 nmol/L ibrutinib or dasatinib or left untreated as a control. After 60 minutes, cell lysates were prepared and kinase binding to Ki-NET beads was assessed using MS. Kinases are grouped by subfamilies. Heatmap shows mean change (log_2_) in kinase binding for ibrutinib- or dasatinib-treated cells relative to control cells for the core 107 kinases derived from three independent experiments. **D,** Graph showing results for ibrutinib for selected kinases, including direct targets (i.e., inhibited by ibrutinib in *in vitro* assays with IC_50_ < 500 nmol/L), kinases which are modulated by ibrutinib but are not direct targets, and kinases which are unaffected by ibrutinib. Graph shows mean (±error) binding normalized to control cells (set to 100%) with statistical significance of differences indicated (Student *t* test; *, *P* < 0.05; **, *P* < 0.01; ****, *P* < 0.0001).

We first examined the ability of kinobeads bearing different broad-specificity KI to capture kinases using the MEC-1 cell line. MEC-1 cells were derived from a patient with CLL undergoing prolymphocytoid transformation (24) and were selected because they have readily detectable levels of constitutively active kinases, including ERK1/2 and AKT (Supplementary Fig. S1A). A schematic workflow for a typical experiment is shown in Fig. 1A.

The most effective kinobeads for kinase capture were those bearing Ki-NET (CTx-0294885), where a core set of 107 kinases were detected in three independent experiments (Fig. 1B and C; Supplementary Data S1). This core set of captured kinases included representatives from all kinase subfamilies and included well-characterized BCR signalosome components such as LYN, SYK, and BTK (Fig. 1C). In contrast, kinobeads bearing bisindolylmaleimide-X (Bis-X), CZC8004, purvalanol-B, pp58, or VI16832 captured substantially fewer kinases (Fig. 1B). Each kinobead also captured various non-kinases and, in some instances, the number of non-kinase targets substantially outweighed the number of kinases (e.g., Bis-X and purvalanol-B). Identified non-kinases may reflect direct capture of non-kinase targets or indirect capture of kinase-associated proteins. Importantly, approximately 70% of the unique kinases identified using any of the six kinobeads were captured using Ki-NET only (Supplementary Fig. S1B) and we therefore focused on Ki-NET kinobeads for further studies. Comparison of intensities determined by MS analysis for the core 107 kinases detected using Ki-NET found a highly significant correlation between the biological repeats for control and inhibitor-treated cells, thereby confirming the reproducibility of the technique (Supplementary Fig. S1C).

We used Ki-NET kinobeads to investigate the effects of ibrutinib and dasatinib on the core set of 107 kinases captured from MEC-1 cells (Fig. 1C). These KIs respectively target BTK and ABL, and were used at concentrations and incubation times that are in line with similar studies describing the *in vitro* effects of these compounds (25). However, both drugs have numerous off-target effects, some of which are shared (25). As expected, the most striking effects of these KIs were observed within the TK and TK-like families (Fig. 1C), where KI occupancy of direct targets explains the reduction in kinase capture. Thus, BTK capture was reduced by >99% in cells treated with ibrutinib and dasatinib (Fig. 1C and D), the latter drug known to also inhibit BTK (26). Other kinases with substantially reduced capture in drug-treated cells included LCK, FYN, LYN, FGR, SRC, CSK, TEC, BLK, and RIPK2, which are known targets of both KIs (25–27).

We also observed more modest reductions in capture of other kinases including ERK1 and 2, PKCβ, and IKKβ (Fig. 1D), kinases which are not known as direct targets of ibrutinib/dasatinib but are likely to be modulated as a response to upstream effects. For example, reduced capture of MEK1 and MEK2 was consistent with the observed reduction in phosphorylation of their substrates, ERK1/2, as detected by immunoblotting (Supplementary Fig. S1). In our system, the steady state expression of kinases was unlikely to be affected by the 60-minute exposure of cells to ibrutinib or dasatinib. Therefore, kinobeads can be used to reveal both the direct occupancy of kinase active site by drug, as well as indirect modulation of active site availability of non-target kinases.

### Kinobead analysis of primary CLL cell signaling

#### Response of CLL kinome to sIgM stimulation

**Figure 2.**
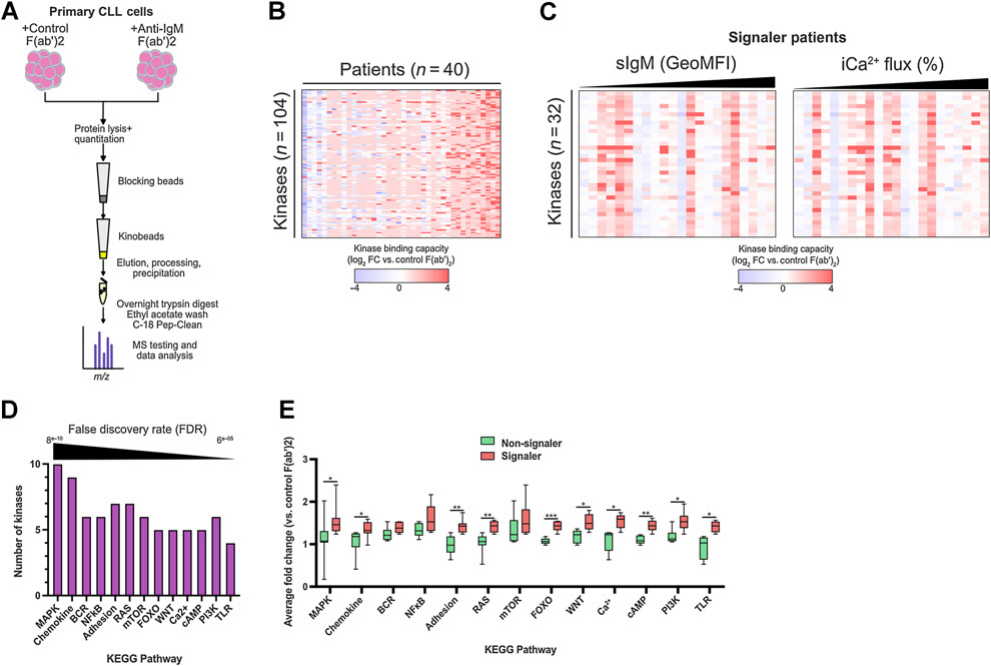
Kinobead profiling of primary CLL cells. **A,** Schematic of the kinobead approach involving primary CLL cells. Protein lysates were prepared from primary cells from patients with CLL (*n* = 40) incubated with control F(ab')_2_ antibody (no stimulation) or anti-IgM. Kinase binding was assessed using Ki-NET beads and MS. **B**, Heatmap showing responses for signature of 104 kinases illustrating relative changes in kinase binding (log_2_ fold change vs. control cells) in response to sIgM stimulation for each patient. **C,** Heatmaps showing signature of 32 kinases for signaler patients. Each patient (columns) was stratified on the basis of either sIgM expression or the proportion of malignant cells capable of iCa^2+^ flux. **D,** Graph derived from STRING analysis of the 32-kinase fingerprint, illustrating the KEGG pathways identified by STRING analysis and the number of kinases from the fingerprint that are involved in each pathway. Pathways are ordered on the basis of FDR. **E,** Graph to show changes in identified KEGG pathways in signaler patients compared with non-signaler patients in response to anti-IgM stimulation. *y*-axis shows on average change in abundance for detected kinases within each KEGG pathway (Student *t* test; *, *P* < 0.05; **, *P* < 0.01; ***, *P* < 0.001).

We next performed Ki-NET kinobead analysis of primary samples from a cohort of 40 patients with CLL. Cells were treated for 5 minutes either with control antibody or with anti-IgM to allow comparison between the “basal” profile signature and that in response to sIgM stimulation, respectively (Fig. 2A). A 5-minute stimulation was chosen to maximize the chance of examining the impact of both proximal kinases and distal effectors. To avoid artefacts associated with extended cell manipulations, we did not purify malignant cells before stimulation/analysis. However, in the samples used, the average proportion of malignant cells was 86% (Table 1; Supplementary Table S1), and T cell–specific kinases, such as ITK, were not detected during our analyses indicating that T-cell contamination minimally affected our assay. In all cases, cell viability was ≥90%. Initial experiments identified 104 kinases from lysates of both control and IgM-stimulated CLL cells (Fig. 2B). Comparison of this signature with that isolated from untreated MEC-1 cells showed, as expected, considerable overlap where 74% of these 104 kinases were common, demonstrating that the kinome fingerprint discovered with MEC-1 can largely be replicated using primary CLL B cells (Supplementary Fig. S2A). Furthermore, the ability of kinobeads to capture kinases appeared independent of their expression level; Nanostring determination of kinase and pseudokinase mRNA levels in CLL cells (Supplementary Fig. S2B; Supplementary Data S2) showed that the major kinase subfamilies identified by both techniques had proportionately similar representation and that individual kinases could be identified by the kinobeads across a wide range of expression.

**Table 1.** Summary information for main patient cohort used within this study (*n* = 40).

Sex	31 Male
	9 Female
Age	Median: 64 (range 42–93)
Stage (Binet)	14 Stage A
	17 Stage B
	9 Stage C
*IGHV* status	15 U-CLL
	17 M-CLL
	8 NA
Karyotype	13 Normal
	13 13qDEL
	6 11qDEL
	2 17pDEL
	2 Complex
	4 NA
Lymphocyte count (×10^9^/L)	Median: 74 (range 19–483)
CLL cells (CD19^+^/CD5^+^; %)	Median: 87 (range 70–94)
Therapy	20 Treatment naïve
	17 FCR
	2 BR
	1 FCR+BR

Abbreviations: BR, bendamustine, rituximab; FCR, fludarabine, cyclophosphamide, rituximab; NA, data not available.

Analysis of the entire cohort showed that capture of some kinases was relatively consistent across all CLL samples, while others were more variably detected. As such, all 104 kinases described were detected in half of the cohort of samples examined. Therefore, we determined a more restricted profile of 32 kinases that were identified as a core fingerprint in lysates from at least 75% of patient samples (Supplementary Data S3). This core fingerprint was largely represented within the kinome fingerprint associated with untreated MEC-1 cells, and with kinome fingerprints associated with sIgM-stimulated MAVER-1 and JeKo-1 cells (Supplementary Fig. S2C). Importantly, key kinases involved in BCR signaling were shared by all cell types used in this comparison, indicating that kinome profiling of CLL cells is able to capture and detect change to the central elements of this pathway.

To probe the reproducibility of our approach using primary cells, biological repeats were carried out. Comparison of MS intensities for control and anti-IgM–treated cells revealed significant correlation across biological repeats (Supplementary Fig. S2D), further confirming the robust nature of the kinobead technique. This allowed observation of the known heterogeneity of sIgM signaling capacity in CLL cells (3, 28), where our assay demonstrates substantial variation in response to anti-IgM between different samples (Fig. 2B and C). Assembly of kinome response into heatmaps revealed how each patient demonstrated a unique profile of response, some samples showed relatively few changes in our identified core fingerprint of kinases following anti-IgM stimulation, whereas others showed much more change. This individual variation was not clearly related to sIgM expression and BCR induction of intracellular calcium flux (iCa^2+^; Fig. 2C), nor was it related to prognostic indicators such as *IGHV* mutation status, Binet staging, or karyotype (Supplementary Fig. S2E). Nevertheless, categorizing patient samples based on their *in vitro* signaling capacity as described previously (ref. 29; i.e., signaler vs. non-signaler, respectfully samples which induce iCa^2+^ flux in response to sIgM stimulation and samples which do not), identified increased capture of SYK and BTK, key kinases in the BCR signaling pathway, in signaler compared with non-signaler patients (Supplementary Fig. S2F). These data suggest that kinobeads measure canonical BCR signaling in CLL cells that is in line with traditional methods.

The individual kinome fingerprints associated with CLL cells from each patient contained a diverse range and number of kinases. This implied the potential for a wide range of signaling networks which could be affected following stimulation of sIgM. To explore this, we next probed the nature of these additional pathways further by applying STRING analysis to the core fingerprint of 32 kinases that were captured by kinobeads. Using a strict FDR (<0.0001) we identified 13 Kyoto Encyclopedia of Genes and Genomes (KEGG) pathways associated with this kinome fingerprint (Fig. 2D), highlighting the shared roles/function of the individual kinases. Reassuringly, the BCR pathway, a key highlight, together with MAPK, mTOR, T-cell receptor (TLR), and NFκB pathways, known to be associated with CLL cell signaling, were identified. Interestingly, other pathways that have been less studied in relation to CLL cell biology, such as those associated with FoxO, RAS, cAMP, and WNT, were also identified. Using this approach, we found that cells from signaler patients demonstrated significant increases in 10/13 (77%) of these KEGG pathways compared with non-signaler patients (Fig. 2E). These findings, taken together with our observations of signaling variability in CLL cells from different patients, suggest the potential of a significantly wider signaling network associated with sIgM activation that involves novel influences that may be individual to the environment or dominant clone of CLL cells within each patient.

#### Kinobeads can be employed to recognize reprogramming of BCR signaling

**Figure 3.**
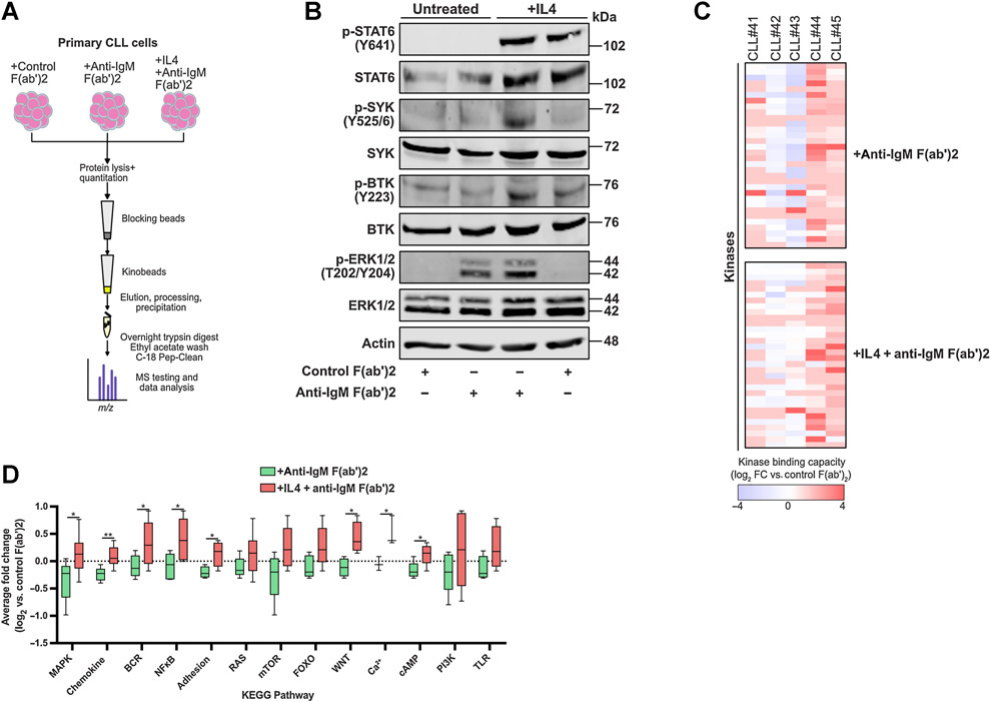
Kinobead-MS can detect reprogramming of sIgM signaling induced through exposure to IL4. **A,** Schematic cartoon illustrating cell conditions/treatments prior to protein lysis and incorporation into the kinobead assay. **B,** Representative immunoblotting (*n* = 2) verifying the action of IL4 pretreatment and anti-IgM stimulation in primary CLL cells. Activation of STAT6, SYK, BTK, and ERK1/2 was measured in cells treated for 5 minutes with 20 μg/mL of either control F(ab')_2_ or anti-IgM F(ab')_2_ (±24 hours pretreatment at 37°C with 10 ng/mL IL4). IL4 treatment alone was used as comparative control. **C,** Heatmaps showing relative changes to kinome signature in a group of 5 patients with CLL stimulated with anti-IgM (±overnight pretreatment with IL4). **D,** Comparison of changes to KEGG pathways (log_2_ scale) within these 5 patients in response to anti-IgM alone or a combination of IL4 and anti-IgM.

We next validated the ability of kinobead profiling to detect modulation of the BCR signaling network in CLL cells. We performed this using CLL cells from 5 signaler patients, incubating them in the presence or absence of IL4 prior to anti-IgM stimulation and lysate collection (Fig. 3A) as described in previous work (22). We confirmed the action of IL4 pretreatment, showing phosphorylation of STAT6 in immunoblotting of treated cell lysates (Fig. 3B). In line with our previous work, such treatment enhanced activation of ERK1/2 as well as SYK and BTK following anti-IgM stimulation (Fig. 3B), suggesting that IL4 changes BCR signaling in CLL cells (22). Kinobead profiling of these cells revealed that such preconditioning resulted in alterations to the kinome fingerprint for each patient (Fig. 3C). Analysis of the KEGG pathways outlined above found that 8/13 (62%) of these were significantly upregulated when cells were treated with both IL4 and anti-IgM (Fig. 3D). These data indicate that kinome profiling can detect the influence of IL4 pretreatment on anti-IgM–induced signaling in CLL cells. Taken together with our data for the main patient cohort, our findings further suggest that not only does the BCR response involve a wider signaling network than previously appreciated, but that this response can be reprogrammed by treating CLL cells with agents such as IL4.

#### Chemoimmunotherapy influences sIgM response in primary CLL

**Figure 4.**
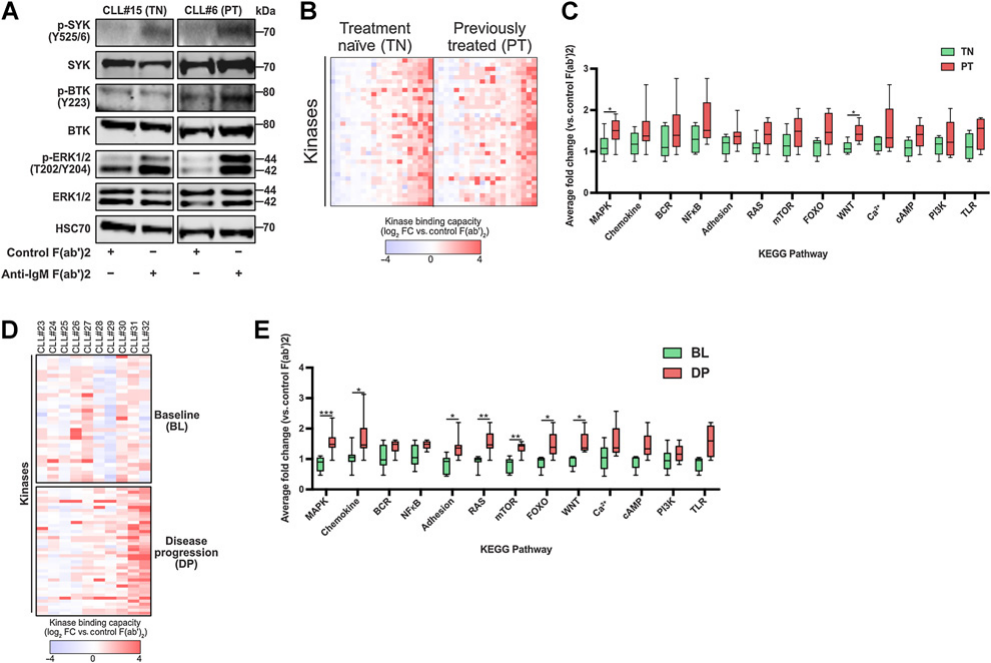
Alterations to signaling pathways induced by therapy can be detected using kinobead-MS. **A,** Immunoblotting, comparing activation of BCR signaling mediators in control and sIgM-stimulated cells between representative treatment-naïve (TN) and previously treated (PT) patients. **B,** Heatmap showing relative changes to kinome capture between signaler patients stratified according to receipt of chemoimmunotherapy. **C,** Comparison of changes to KEGG pathways associated with our kinome signature between the TN and PT patients used to prepare part B. **D,** Heatmap comparing sIgM activation kinome fingerprints for ARCTIC/ADMIRE trial patients (*n* = 10) using their baseline (pre-therapy) sample and their disease progression (DP) sample. **E,** Graph comparing changes in KEGG pathways between BL and DP patients used to prepare part D.

CLL cell heterogeneity between patient samples results not only from their biology and clinical presentation, but also from patient receipt of therapy. This latter aspect, as in other tumor types, may cause alterations in signaling within malignant cells. To explore this possibility in CLL, we initially examined well-established mediators of BCR signaling using immunoblotting (Fig. 4A). These found that upon stimulation of sIgM, kinases such as SYK, BTK, and ERK1/2 demonstrated increased activation within cells from previously treated patients (PT) compared with those who were treatment naïve (TN). To explore this further, we stratified kinobead-MS data from signaler patients within our cohort based upon receipt of chemoimmunotherapy. We found that although the heatmaps corresponding to CLL cell responses from TN patients and from PT patients were similar (Fig. 4B), analysis of our identified KEGG pathways showed PT patients displayed increased changes in all 13, which in the case of MAPK and WNT were significantly different (*P* < 0.05; Student *t* test; Fig. 4C). Importantly, these alterations of the kinome were not due to differential kinase expression because comparison of kinome mRNA levels in TN and PT CLL cells found a highly significant level of correlation (*P* < 0.0001; Supplementary Fig. S3A), nor were they due to changes in phosphatase activity (Supplementary Fig. S3B and S3C) because we were unable to observe any differences in the expression of key B-cell phosphatases (30–34).

Within our original 40-patient cohort, we examined the kinome responses of disease progression (DP) samples donated by patients recruited to the FCR (fludarabine, cyclophosphamide, rituximab) arm of the ARCTIC/ADMIRE clinical trials. To confirm the influence of chemoimmunotherapy on sIgM-induced signaling in CLL cells, we next performed kinobead analysis on the respective matched baseline (BL) samples from these patients. The unique, patient-specific nature of kinome responses upon anti-IgM stimulation was once again apparent in the BL samples (Fig. 4D), and these individual kinome responses completely changed in the matched patient sample taken at DP. KEGG pathway analysis of this change found that seven of our identified pathways, including MAPK, RAS, mTOR, chemokine, adhesion, FOXO, and WNT, were significantly upregulated in CLL cells taken at DP compared with those taken at BL (Fig. 4E). Collectively, these data demonstrate BCR signal rewiring in CLL cells suggesting that this is an adaptive response of the malignant cells in a resistance mechanism to chemoimmunotherapy.

#### CLL cells adapt BCR-induced signaling in response to ibrutinib therapy *in vivo*

**Figure 5.**
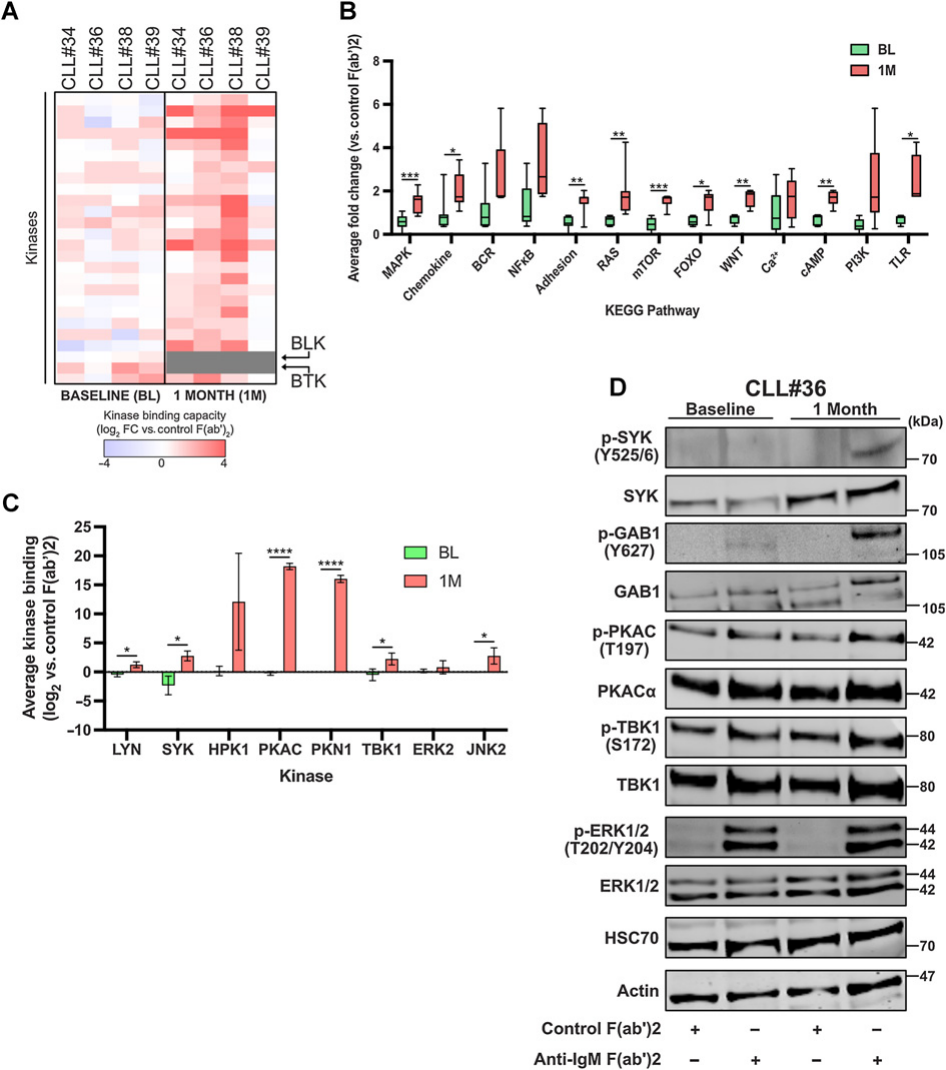
Longitudinal kinobead profiling of sIgM response in patients undergoing ibrutinib therapy. **A,** Heatmap comparing sIgM activation kinome fingerprints for IcICLLe clinical trial patients (*n* = 4) using their baseline (pre-therapy) sample and samples taken 1 month following commencement of ibrutinib therapy. Grey boxes denote loss of kinase binding. **B,** Graph comparing changes in KEGG pathways between baseline (BL) and 1-month (1M) samples. **C,** Graph comparing relative change of binding for kinases found to demonstrate significant change within 1-month samples in response to sIgM stimulation. **D,** Representative immunoblotting corroborating changes to kinases found by kinobead-MS.

To investigate whether change in BCR signaling occurs in CLL cells from patients on other therapies we performed kinome profiling on four patient samples from the IcICLLe clinical trial testing ibrutinib. Here we compared anti-IgM response in CLL cells taken at baseline (pre-ibrutinib) and again at 1 month following initiation of ibrutinib (Fig. 5A). We identified a reduced signature of 26 kinases which was common between CLL cells taken at both timepoints and this signature showed dramatic change in samples from patients receiving ibrutinib in a way that suggested increased signal intensity (Fig. 5A). This increase was not related sample by sample to lymphocyte count but could correlate to sIgM expression on the malignant cells in a way that is in line with previous work (35), showing that circulating CLL cells from patients undergoing ibrutinib therapy demonstrated increased sIgM levels (Supplementary Fig. S4A). Importantly, in the ibrutinib-treated samples, there was a loss of BTK and BLK from the fingerprint (Fig. 5A), despite their presence within the input lysate, as shown by immunoblotting of BTK (Supplementary Fig. S4B). This loss of BTK binding to Ki-NET was confirmed using immunoblotting of lysates derived from cells from patients undergoing ibrutinib therapy within our clinics (Supplementary Fig. S4C) and illustrated the *in vivo* action of ibrutinib to block access of BTK, and also of BLK because ibrutinib targets both these kinases in the same way (27), to the Ki-NET beads. Intriguingly, it was possible to recognize ERK1/2 activation was maintained within ibrutinib-treated cells, implying that cells retain the capacity to induce downstream effectors critical to tumor cell biology. Application of KEGG pathway analysis showed that nine of our identified pathways were significantly enhanced in CLL cells from ibrutinib-treated patient samples. In particular, the finding of enhanced TLR pathway signaling is consistent with those of others who have described that such signaling is partially resistant to ibrutinib (36).

Although not significant, there seemed a distinct increase in BCR pathway signaling despite the loss of BTK in stimulated CLL cells from patients receiving ibrutinib. To explore this enhanced signaling further, we examined kinobead-MS data to identify possible mediators/regulators. Consistent with our observations of enhanced BCR pathway signaling, we found significantly increased representation of LYN, SYK, and JNK2 within kinome profiles of CLL cells from ibrutinib-treated patients. However, these same profiles also showed significantly increased representation of other potential drivers of signaling, notably PKACα, PKN1, and TBK1 (Fig. 5C). Immunoblotting of baseline and 1-month samples verified our kinobead-MS finding of enhanced activation of these kinases (Fig. 5D). Levels of phospho-SYK, -GAB1 (a reported downstream effector of SYK), -PKACα, and -TBK1 were all found enhanced in anti-IgM–stimulated CLL cells from ibrutinib-treated patients. Taken together, these data suggest a compensatory mechanism of signal pathway reprogramming in CLL cells is activated in patients within a month of receiving ibrutinib therapy.

## Discussion

In the current study, we employed a kinobead-based approach to examine BCR signaling within primary CLL cells. The results we generate are, to the best of our knowledge, the most comprehensive proteomic profiling of BCR signaling in these cells. An important and novel finding we make is adaptation in sIgM signaling brought about through receipt of therapy. Thus, we show that CLL cells from patients who had received either traditional chemoimmunotherapy or ibrutinib change their response to BCR engagement. Our analyses further suggest new drivers of signaling within the BCR pathway become involved. Our study therefore creates new understanding of the role BCR signaling plays within the natural history of CLL.

The most common method for investigating signaling in CLL involves the use of antibodies to screen restricted numbers of kinases and phosphoprotein targets using immunoblotting or flow cytometry. While informative, these methods lack the resolution required to observe widespread changes to signaling mediators within cells in response to various conditions. Alternatively, MS is an emerging technology in terms of analyzing signaling in CLL cells, used so far only to provide a global view of protein expression to investigate malignant cell biology and its relation to disease severity (37–39). However, these experiments typically use in excess of 10^8^ CLL cells, limiting both their application to patient samples with high cell numbers and the number of interventions that can be performed. In our approach we used a kinobead-based protocol to isolate signaling proteins that could then be identified by MS to ultimately examine changes to sIgM signaling in CLL cells. This method reproducibly isolated a profile of over 100 kinases from MEC-1 cells. Because this cell line cannot respond to sIgM cross-linking, it was used as a model cell line to establish the parameters of the kinome assay for use with primary CLL cells. The results we generated from MEC-1 and CLL cells represent good coverage of all major kinase subfamilies expressed in these cells. Importantly, the numbers of CLL cells used in our experiments is considerably lower (10^7^) than that used in other proteomic studies using CLL cells (37–39), but kinobeads were still able to detect a substantial proportion of the kinome in these cells, a finding that is consistent with kinome profiling studies on other cell types (13–16). Thus, kinobead extraction of signaling proteins provides an efficient means to accurately assess malignant cell response in samples derived from patients, CLL or other haemic malignancy, enrolled in clinical trials testing therapy.

Our analysis of response to sIgM engagement in primary CLL cells showed that the fingerprint of activated kinases from individual samples varied widely. Such signaling heterogeneity will be related to the acknowledged variability of CLL cell response to BCR engagement (3, 28, 29) and our data provide new insight into this phenomenon through identification of 13 KEGG pathways that show change in the kinome signatures between signaler and non-signaler cells. This change is likely linked to disease outcome owing to the demonstrated relationship of signaler and non-signaler phenotypes with disease outcome in CLL (6). While some of these pathways are readily recognized as being associated with the pathobiology of CLL, some are less well known and reveal potential new drivers of signaling within the BCR pathway. Our data show that these drivers are particularly important for CLL cell adaptation of BCR signaling in response to therapy. We applied kinome profiling to matched patient samples taken at baseline and then again following disease progression from clinical trials testing the efficacy of FCR treatment. We found that 6 of the 13 identified KEGG pathways showed significant change between baseline and disease progression. In a similar way, analysis of matched samples from the IcICLLe clinical trial testing the efficacy of ibrutinib revealed that 9 of the 13 identified KEGG pathways had significant change between samples taken at baseline and after 1 month of therapy. Taken together, these data suggest a novel finding highlighting reprogramming of the BCR signaling pathway can take place in CLL cells in response to therapy. The notion that BCR signaling can reprogram is strongly supported by the reprogramming effect IL4 has on BCR signaling in CLL cells demonstrated within a study of BCR-induced Ca^2+^-flux (22) and from our kinome profiling experiments shown in Fig. 3. Such reprogramming might be an expected response of CLL cells in patients on ibrutinib therapy; similar reprogramming was observed in (AZD6244)-treated triple-negative breast cancer cells where kinobead analysis has demonstrated adaptive activation of receptor tyrosine kinases in response to MEK inhibition (13), and in other malignant cell systems and small molecule agents (40, 41). A definitive demonstration relating BCR signal reprogramming and ibrutinib resistance will need further investigation. However, our data describe the novel finding that signal pathway reprogramming is a potential resistance mechanism for CLL cells and cytotoxic therapies, a finding that is particularly exciting for our understanding of the role BCR engagement plays in the pathophysiology of this disease.

An intriguing aspect of our analysis is the identification of kinase nodes that could act to drive the adaptive potential of sIgM signaling in response to therapy. This is best defined in the context of studying the impact of ibrutinib. Despite the clear loss of BTK activity in samples taken 1 month following commencement of therapy, we detected significant increases in SYK, LYN, JNK2, PKACα, PKN1, and TBK1 representation within kinome profiles of sIgM-stimulated CLL cells. Moreover, we also detected marked increases in ERK2 and HPK1 representation as well. Roles for PKACα and PKN1 in CLL cells are unclear but is implied that the latter is involved in lymphocyte migration (42).

Such findings directly implicate the capacity of primary CLL cells to retain their ability to respond to antigen engagement in the presence of a covalent inhibitor of BTK (a key mediator of the BCR signal pathway) by modifying signaling to induce other downstream effectors critical to tumor biology. Our data strongly indicates SYK involvement in this process as TBK1, and HPK1 have been shown to interact downstream of activated SYK (42–45). Moreover, our demonstration of increased GAB1^Y627^ phosphorylation in BCR-stimulated CLL cells from ibrutinib-treated patients suggests that more proximal effectors become involved when BTK activity is lost. GAB1 is a known adapter of BCR signaling and is a target of SYK that is recognized as an amplifier of BCR signaling (46, 47). When phosphorylated at Y627, GAB1 activates SHP2 which subsequently facilitates activation of ERK1/2. Therefore, our data suggest a potential molecular mechanism by which primary CLL cells can adapt to ibrutinib that is employed quickly following initiation of therapy. In this manner, there is the possibility to characterize the evolution of BCR signaling within malignant B cells as they adapt to treatment, and whether this ultimately correlates to the clinical course of patients. It was not possible to demonstrate this within the current study due to the number of patients with the cohort; however, this represents a key area of investigation in the future.

A unique feature of kinome profiling is the ability to analyze drug occupancy in primary CLL cells from patients receiving ibrutinib therapy. Ibrutinib covalently modifies BTK at Cys^(481)^ and is therefore irreversibly bound to this protein where it blocks access to ATP and compounds that mimic ATP. We show that the ability of the inhibitor beads to isolate BTK and other tyrosine kinases where the Cys residue is in a relevant position (i.e., BLK) to be modified by ibrutinib is eliminated in samples taken from patients on this type of therapy. Previous studies of hematologic cells using a kinobead approach have purely investigated drug–protein interactions (48–50). Our study shows that kinobeads are also a novel molecular tool to investigate drug dosing in patients whereby the minimal dose of ibrutinib, or similar drug, can be determined that still affects BTK function. Such knowledge is useful in the quest to reduce/remove unwanted side effects.

CLL is a disease that is incurable because of the relative ease with which the malignant cells develop resistance to therapy (1). Until now, research into the mechanisms involved in this resistance have focused on the evolution of genetic and epigenetic clones, and on the role the microenvironment plays in promoting survival of these clones (1). The data presented in this article support a further mechanism of resistance involving rewiring of BCR signaling that is central to the pathogenesis of CLL. Our demonstration that the kinome response induced by BCR engagement changes in CLL cells when patients relapse from chemoimmunotherapy has important implications for the use of second-line treatments involving KIs. Future work is now needed to compare the signal pathway reprogramming our data reveal is activated in CLL cells from patients receiving ibrutinib therapy. Considering the overwhelming beneficial impact of ibrutinib in terms of survival when compared with chemoimmunotherapy (51), such work will ultimately provide guidance to clinicians and establish this KI as a front-line therapy in the treatment of patients with CLL.

## Authors' Disclosures

A.J. Linley reports grants from Leuka and North West Cancer Research Fund during the conduct of the study. L.I. Karydis reports grants from Kay Kendall Leukaemia Fund during the conduct of the study. A.R. Pettitt reports non-financial support and other support from Celgene; grants and non-financial support from GSK/Novartis and Chugai; grants, non-financial support, and other support from Roche; personal fees from AbbVie; grants from Napp; and grants, personal fees, and other support from Gilead outside the submitted work. A.C. Rawstron reports grants and personal fees from Abbvie, Celgene, Janssen, Pharmacyclics, and Roche during the conduct of the study; A.C. Rawstron also reports non-financial support from Beckman Coulter and personal fees, non-financial support, and other support from Becton Dickinson outside the submitted work. P. Hillmen reports grants and personal fees from Janssen, Pharmacyclics, AbbVie, and Roche; grants from Gilead; and personal fees from AstraZeneca outside the submitted work. A.J. Steele reports grants from Kay Kendall Leukaemia Fund during the conduct of the study. J.R. Slupsky reports grants from Northwest Cancer Research Fund, Kay Kendal Leukaemia Fund, and Royal Embassy of Saudi Arabia Cultural Bureau during the conduct of the study, as well as grants from Netherlands Translational Research Centre and Verastem Oncology outside the submitted work. No disclosures were reported by the other authors.

## Supplementary Material

Supplementary Legend Supplementary Legend

Supplementary Figure 1 Supplementary Figure 1: A; Immunoblot analysis of MEC-1 cells showing expression of total and phospho-ERK1/2 and AKT in untreated control (NA), ibrutinib or dasatinib-pre-treated cells (both at 500 nM for 60 minutes). Actin was analyzed as an additional loading control. B; Venn diagrams showing overlap of kinases isolated by the different KI used to create kinobeads during this study. C; Correlation graphs comparing intensities (Log10 scale) for kinases isolated from MEC-1 lysates by Ki-NET beads in untreated, ibrutinib-and dasatinib pre-treated cells.

Supplementary Figure 2 Supplementary Figure 2: A; Venn diagram showing overlap of larger kinobead signatures derived from MEC-1 and primary CLL cell experiments. B; Graph comparing kinases identified at either mRNA level, protein (by kinobead isolation) or both in our patient cohort. C; Venn diagram to compare overlap of the refined 32 kinase signature gained from primary CLL experiments in relation to the malignant lymphoid cell lines MEC-1, MAVER-1 and JeKo-1. D; Correlation graphs for 2 representative CLL patients, comparing intensities gained for our kinome signature for 2 biological repeats for baseline (Control F(ab')2) cells and in response to anti-IgM treatment. E; Heatmaps showing kinome fingerprints for CLL patients stratified according to IGHV mutation status, Binet staging or karyotype status. F; Graphs illustrating relative change in isolation of SYK and BTK in primary CLL cells from patients stratified according to in vitro iCa2+ flux response as being Non-Signaler (NS; iCa2&lt;5%), or Signaler (S; iCa2+&gt;5%).

Supplementary Figure 3 Supplementary Figure 3: A; Correlation of kinase mRNA expression determined by Nanostring between treatment naïve (TN) and previously treated (PT) patients. B; Flow cytometric analysis of inhibitory coreceptors and C; immunoblot analysis of phosphatases in three samples from untreated (UT) patients and three samples from previously treated (PT) patients. In B, graph shows results for individual samples and mean ({plus minus}error). In C, GAPDH was analyzed as an additional loading control.

Supplementary Figure 4 Supplementary Figure 4: A; Relative changes to surface IgM (sIgM) (GeoMFI) expression for 4 IcICLLe clinical trial patients between baseline and 1-month after initial receipt of ibrutinib treatment. B; Representative immunoblotting confirming presence of BTK within the input lysate of the baseline (BL) and 1-month (1M) treatment samples from a patient recruited to the IcICLLe clinical trial. C; Immunoblotting to illustrate loss of BTK binding to kinobeads through in vivo action of ibrutinib. Kinobead isolation was performed on matched samples for 2 representative patients, comparing the baseline sample to that taken 1-month following commencement of ibrutinib treatment. Kinobead elutions were mixed with loading buffer and separated by SDS-PAGE prior to blotting and probing.

Supplementary Dataset 1 Large kinome signature gained from experiments using MEC-1 cells, showing changes in response to ibrutinib and dasatinib treatment.

Supplementary Dataset 2 Intensities for kinases detected from Nanostring experiments involving primary CLL cells.

Supplementary Dataset 3 Refined kinome signature ratio data gained from experiments involving primary CLL cells from a cohort of 40 patients.

Supplementary Table 1 Characteristic data for primary CLL cells from to main 40 patient cohort.

## References

[1] Kipps TJ, Stevenson FK, Wu CJ, Croce CM, Packham G, Wierda WG, et al Chronic lymphocytic leukaemia. Nat Rev Dis Primers 2017;19:16096. 10.1038/nrdp.2016.96 PMC5336551 28102226

[2] Burger JA, Wiestner A. Targeting B cell receptor signaling in cancer: preclinical and clinical advances. Nat Rev Cancer 2018;18:148–67. 29348577 10.1038/nrc.2017.121

[3] Stevenson FK, Krysov S, Davies AJ, Steele AJ, Packham G. B-cell receptor signaling in chronic lymphocytic leukemia. Blood 2011;118:4313–20. 21816833 10.1182/blood-2011-06-338855

[4] Dühren-von Minden M, Übelhart R, Schneider D, Wossning T, Bach MP, Buchner M, et al Chronic lymphocytic leukaemia is driven by antigen independent cell-autonomous signaling. Nature 2012;489:309–12. 22885698 10.1038/nature11309

[5] Minici C, Gounari M, Übelhart R, Scarfó L, Dühren-von Minden M, Schneider D, et al Distinct homotypic B-cell receptor interactions shape the outcome of chronic lymphocytic leukaemia. Nat Commun 2017;8:15746. 28598442 10.1038/ncomms15746 PMC5472768

[6] D'Avola A, Drennan S, Tracy I, Henderson I, Chiecchio L, Larrayoz M, et al Surface IgM expression and function are associated with clinical behavior, genetic abnormalities, and DNA methylation in CLL. Blood 2016;128:816–26. 27301861 10.1182/blood-2016-03-707786

[7] Alinari L, Quinion C, Blum KA. Bruton's tyrosine kinase inhibitors in B-cell non-Hodgkin's lymphomas. Clin Pharmacol Ther 2015;97:467–77. 10.1002/cpt.65 25670208

[8] Byrd JC, Furman RR, Coutre SE, Burger JA, Blum KA, Coleman M, et al Three year follow up of treatment-naïve and previously treated patients with CLL and SLL receiving single agent ibrutinib. Blood 2015;125:2497–506. 25700432 10.1182/blood-2014-10-606038 PMC4400288

[9] O'Brien S, Jones JA, Coutre SE, Mato AR, Hillmen P, Tam C, et al Ibrutinib for patients with relapsed or refractory chronic lymphocytic leukaemia with 17p deletion (RESONATE-17): a phase 2, open-label, multicentre study. Lancet Oncol 2016;17:1409–18. 27637985 10.1016/S1470-2045(16)30212-1

[10] Furman RR, Cheng S, Lu P, Setty M, Perez AR, Guo A, et al Ibrutinib resistance in chronic lymphocytic leukemia. N Engl J Med 2014;370:2352–4. 24869597 10.1056/NEJMc1402716 PMC4512173

[11] Woyach JA, Furman RR, Liu TM, Ozer HG, Zapatka M, Ruppert AS, et al Resistance mechanisms for the Bruton's tyrosine kinase inhibitor ibrutinib. N Engl J Med 2014;370:2286–94. 24869598 10.1056/NEJMoa1400029 PMC4144824

[12] Wist M, Meier L, Gutman O, Haas J, Endres S, Zhou Y, et al Noncatalytic Bruton's tyrosine kinase activates PLCg2 variants mediating ibrutinib resistance in human chronic lymphocytic leukemia cells. J Biol Chem 2020;295:5717–36. 32184360 10.1074/jbc.RA119.011946 PMC7186163

[13] Duncan JS, Whittle MC, Nakamura K, Abell AN, Midland AA, Zawistowski JS, et al Dynamic reprogramming of the kinome in response to targeted MEK inhibition in triple-negative breast cancer. Cell 2012;149:307–21. 22500798 10.1016/j.cell.2012.02.053 PMC3328787

[14] Kurimchak AM, Shelton C, Duncan KE, Johnson KJ, Brown J, O'Brien S, et al Resistance to BET bromodomain inhibitors is mediated by kinome reprogramming in ovarian cancer. Cell Rep 2016;16:1273–86. 27452461 10.1016/j.celrep.2016.06.091 PMC4972668

[15] Mundt F, Rajput S, Li S, Ruggles KV, Mooradian AS, Mertins P, et al Mass-spectrometry-based proteomics reveals potential roles of NEK9 and MAP2K4 in resistance to PI3K inhibition in triple-negative breast cancers. Cancer Res 2018;78:2732–46. 29472518 10.1158/0008-5472.CAN-17-1990 PMC5955814

[16] Palve V, Kuenzi BM, Rix U. Unraveling the rewired network. Nat Chem Biol 2018;14:746–7. 29942077 10.1038/s41589-018-0083-7

[17] Bantscheff M, Eberhard D, Abraham Y, Bastuck S, Boesche M, Hobson S, et al Quantitative chemical proteomics reveals mechanisms of action of clinical ABL kinase inhibitors. Nat Biotechnol 2007;25:1035–44. 17721511 10.1038/nbt1328

[18] Joseph RE, Wales TE, Fulton DB, Engen JR, Andreotti AH. Achieving a graded immune response: BTK adopts a range of active/inactive conformations dictated by multiple interdomain contacts. Structure 2017;25:1481–94. 28867612 10.1016/j.str.2017.07.014 PMC5629114

[19] Zhang F, Strand A, Robbins D, Cobb MH, Goldsmith EJ. Atomic structure of the MAP kinase ERK2 at 2.3 A resolution. Nature 1994;367:704–11. 8107865 10.1038/367704a0

[20] Donnella HJ, Webber JT, Levin RS, Camarda R, Momcilovic O, Bayani N, et al Kinome rewiring reveals AURKA limits PI3K-pathway inhibitor efficacy in breast cancer. Nat Chem Biol 2018;14:768–77. 29942081 10.1038/s41589-018-0081-9 PMC6051919

[21] Krysov S, Dias S, Paterson A, Mockridge CI, Potter KN, Smith KA, et al Surface IgM stimulation induces MEK1/2-dependent MYC expression in chronic lymphocytic leukemia. Blood 2012;119:170–9. 22086413 10.1182/blood-2011-07-370403

[22] Aguilar-Hernandez MM, Blunt MD, Dobson R, Yeomans A, Thirdborough S, Larrayoz M, et al IL-4 enhances expression and function of surface IgM in CLL cells. Blood 2016;127:3015–25. 27002119 10.1182/blood-2015-11-682906

[23] Goodman JK, Zampronio CG, Jones AME, Hernandez-Fernaud JR. Updates of the in-gel digestion method for protein analysis by mass spectrometry. Proteomics 2018;18:e1800236. 30259661 10.1002/pmic.201800236 PMC6492177

[24] Stacchini A, Aragno M, Vallario A, Alfarano A, Circosta P, Gottardi D, et al MEC1 and MEC2: two new cell lines derived from B-chronic lymphocytic leukaemia in prolymphocytoid transformation. Leuk Res 1999;23:127–36. 10071128 10.1016/s0145-2126(98)00154-4

[25] Wilson LJ, Linley A, Hammond DE, Hood FE, Coulson JM, MacEwan DJ, et al New perspectives, opportunities, and challenges in exploring the human protein kinome. Cancer Res 2018;78:15–29. 29254998 10.1158/0008-5472.CAN-17-2291

[26] Hantschel O, Rix U, Schmidt U, Bürkstümmer T, Kneidinger M, Schütze G, et al The Btk tyrosine kinase is a major target of the Bcr-Abl inhibitor dasatinib. Proc Natl Acad Sci U S A 2007;104:13283–8. 17684099 10.1073/pnas.0702654104 PMC1940229

[27] Kim E, Hurtz C, Koehrer S, Wang Z, Balasubramanian S, Chang BY, et al Ibrutinib inhibits pre-BCR^+^ B-cell acute lymphoblastic leukemia progression by targeting BTK and BLK. Blood 2017;129:1155–65. 28031181 10.1182/blood-2016-06-722900 PMC5374732

[28] Packham G, Stevenson F. The role of the B-cell receptor in the pathogenesis of chronic lymphocytic leukaemia. Semin Cancer Biol 2010;20:391–9. 20816790 10.1016/j.semcancer.2010.08.004

[29] Mockridge CI, Potter KN, Wheatley I, Neville LA, Packham G, Stevenson FK. Reversible anergy of sIgM-mediated signaling in the two subsets of CLL defined by VH-gene mutational status. Blood 2007;109:4424–31. 17255355 10.1182/blood-2006-11-056648

[30] Gabelloni ML, Borge M, Galletti J, Cañones C, Calotti PF, Bezares RF, et al SHIP-1 protein level and phosphorylation status differs between CLL cells segregated by ZAP-70 expression. Br J Haematol 2008;140:117–9. 18005265 10.1111/j.1365-2141.2007.06891.x

[31] Zhu JW, Brdicka T, Katsumoto TR, Lin J, Weiss A. Structurally distinct phosphatases CD45 and CD148 both regulate B cell and macrophage immunoreceptor signaling. Immunity 2008;28:183–96. 18249142 10.1016/j.immuni.2007.11.024 PMC2265106

[32] Wu HJ, Bondada S. CD72, a coreceptor with both positive and negative effects on B lymphocyte development and function. J Clin Immunol 2009;29:12–21. 19067131 10.1007/s10875-008-9264-6

[33] Tibaldi E, Brunati AM, Zonta F, Frezzato F, Gattazzo C, Zambello R, et al Lyn-mediated SHP-1 recruitment to CD5 contributes to resistance to apoptosis of Bcell chronic lymphocytic leukemia cells. Leukemia 2011;25:1768–81. 21701493 10.1038/leu.2011.152

[34] Sieger N, Fleischer SJ, Mei HE, Reiter K, Shock A, Burmester GR, et al CD22 ligation inhibits downstream B cell receptor signaling and Ca (2+) flux upon activation. Arthritis Rheum 2013;65:770–9. 23233360 10.1002/art.37818

[35] Drennan S, Chiodin G, D'Avola A, Tracy I, Johnson PW, Trentin L, et al Ibrutinib therapy releases leukemic surface IgM from antigen drive in chronic lymphocytic leukemia patients. Clin Cancer Res 2019;25:2503–12. 30373751 10.1158/1078-0432.CCR-18-1286

[36] Dadashian EL, McAuley EM, Liu D, Shaffer AL 3rd, Young RM, Iyer JR, et al TLR signaling is activated in lymph node-resistant CLL cells and is only partially inhibited by ibrutinib. Cancer Res 2019;79:360–71. 30498085 10.1158/0008-5472.CAN-18-0781 PMC6342512

[37] Kashuba E, Eagle GE, Bailey J, Evans P, Welham KJ, Allsup D, Cawkwell L. Proteomic analysis of B-cell receptor signaling in chronic lymphocytic leukaemia reveals a possible role for kininogen. J Proteomics 2013;91:478–85. 23938224 10.1016/j.jprot.2013.08.002

[38] Eagle GE, Zhuang J, Jenkins RE, Till KJ, Jithesh PV, Lin K, et al Total proteome analysis identifies migration defects as a major pathogenic factor in immunoglobulin heavy chain variable region (IGHV)-unmutated chronic lymphocytic leukemia. Mol Cell Proteomics 2015;14:933–45. 25645933 10.1074/mcp.M114.044479 PMC4390271

[39] Johnston HE, Carter MJ, Larrayoz M, Clarke J, Garbis SD, Oscier D, et al Proteomics profiling of CLL versus healthy B-cells identifies putative therapeutic targets and a subtype-independent signature of spliceosome dysregulation. Mol Cell Proteomics 2018;17:776–91. 29367434 10.1074/mcp.RA117.000539 PMC5880099

[40] Johnson GL, Stuhlmiller TJ, Angus SP, Zawistowski JS, Graves LM. Molecular pathways: adaptive kinome reprogramming in response to targeted inhibition of the BRAF-MEK-ERK pathway in cancer. Clin Cancer Res 2014;20:2516–22. 24664307 10.1158/1078-0432.CCR-13-1081 PMC4024346

[41] Stuhlmiller TJ, Miller SM, Zawistowski JS, Nakamura K, Beltran AS, Duncan JS, et al Inhibition of lapatinib-induced kinome reprogramming in ERBB2positive breast cancer by targeting BET family bromodomains. Cell Rep 2015;11:390–404. 25865888 10.1016/j.celrep.2015.03.037 PMC4408261

[42] Mashud R, Nomachi A, Hayakawa A, Kubouchi K, Danno S, Hirata T, et al Impaired lymphocyte trafficking in mice deficient in the kinase activity of PKN1. Sci Rep 2017;7:7663. 28794483 10.1038/s41598-017-07936-9 PMC5550459

[43] Liou J, Kiefer F, Dang A, Hashimoto A, Cobb MH, Kurosaki T, Weiss A. HPK1 is activated by lymphocyte antigen receptors and negatively regulates AP-1. Immunity 2000;12:399–408. 10795738 10.1016/s1074-7613(00)80192-2

[44] Tsuji S, Okamoto M, Yamada K, Okamoto N, Goitsuka R, Arnold R, et al B cell adapter containing src homology 2 domain (BASH) links B cell receptor signaling to the activation of hematopoietic progenitor kinase 1. J Exp Med 2001;194:529–39. 11514608 10.1084/jem.194.4.529 PMC2193495

[45] Lin YC, Huang DY, Chun CL, Lin YL, Lin WW. The tyrosine kinase Syk differentially regulates Toll-like receptor signaling downstream of the adapter molecules TRAF6 and TRAF3. Sci Signal 2013;6:ra71. 23962979 10.1126/scisignal.2003973

[46] Ingham RJ, Santos L, Dang-Lawson M, Holgado-Madruga M, Dudek P, Maroun CR, et al The Gab1 docking protein links Bcell antigen receptor to the phosphatidylinositol 3-kinase/Akt signaling pathway and to the SHP2 tyrosine phosphatase. J Biol Chem 2001;276:12257–65. 11278704 10.1074/jbc.M010590200

[47] Seda V, Mraz M. B-cell receptor signalling and its crosstalk with other pathways in normal and malignant cells. Eur J Haematol 2015;94:193–205. 25080849 10.1111/ejh.12427

[48] Kruse U, Pallasch CP, Bantscheff M, Eberhard D, Frenzel L, Ghidelli S, et al Chemoprotemics-based kinome profiling and target deconvolution of clinical multi-kinase inhibitors in primary chronic lymphocytic leukemia cells. Leukemia 2011;25:89–100. 20944678 10.1038/leu.2010.233

[49] Dittus L, Werner T, Muelbaier M, Bantscheff M. Differential kinobeads profiling for target identification of irreversible kinase inhibitors. ACS Chem Biol 2017;12:2515–21. 28876896 10.1021/acschembio.7b00617

[50] Eberl HC, Werner T, Reinhard FB, Lehmann S, Thomson D, Chen P, et al Chemical proteomics reveals target selectivity of chemical Jak inhibitors in human primary cells. Sci Rep 2019;9:14159. 31578349 10.1038/s41598-019-50335-5 PMC6775116

[51] Woyach JA, Ruppert AS, Heerema NA, Zhao W, Booth AM, Ding W, et al Ibrutinib alone or in combination with rituximab produces superior progression free survival (PFS) compared with bendamustine plus rituximab in untreated older patients with chronic lymphocytic leukemia (CLL): Results of Alliance North America Intergroup Study A041202. Blood 2018;132:6.

