# Supplementary material for: Kinobead Profiling Reveals Reprogramming of BCR Signaling in Response to Therapy within Primary CLL Cells
Source: Clin Cancer Res. 2021 Aug 11;27(20):5647–59. doi: 10.1158/1078-0432.CCR-21-0161 (PMC9662893; doi:10.1158/1078-0432.CCR-21-0161)
Supplement: Supplementary Legend [file 10780432ccr210161-sup-258762_3_supp_7293100_qx2d42.docx]

# Supplementary Figure Legends

**Supplementary Figure 1**: **A**; Immunoblot analysis of MEC-1 cells showing expression of total and phospho-ERK1/2 and AKT in untreated control (NA), ibrutinib or dasatinib-pre-treated cells (both at 500 nM for 60 minutes). Actin was analyzed as an additional loading control. **B**; Venn diagrams showing overlap of kinases isolated by the different KI used to create kinobeads during this study. **C**; Correlation graphs comparing intensities (Log10 scale) for kinases isolated from MEC-1 lysates by Ki-NET beads in untreated, ibrutinib-and dasatinib pre-treated cells.

**Supplementary Figure 2: A**; Venn diagram showing overlap of larger kinobead signatures derived from MEC-1 and primary CLL cell experiments. **B**; Graph comparing kinases identified at either mRNA level, protein (by kinobead isolation) or both in our patient cohort. **C**; Venn diagram to compare overlap of the refined 32 kinase signature gained from primary CLL experiments in relation to the malignant lymphoid cell lines MEC-1, MAVER-1 and JeKo-1. **D**; Correlation graphs for 2 representative CLL patients, comparing intensities gained for our kinome signature for 2 biological repeats for baseline (Control F(ab’)2) cells and in response to anti-IgM treatment. **E**; Heatmaps showing kinome fingerprints for CLL patients stratified according to *IGHV* mutation status, Binet staging or karyotype status. **F**; Graphs illustrating relative change in isolation of SYK and BTK in primary CLL cells from patients stratified according to *in vitro* iCa^2+^ flux response as being Non-Signaler (NS; iCa^2^<5%), or Signaler (S; iCa^2+^>5%).

**Supplementary Figure 3:** **A**; Correlation of kinase mRNA expression determined by Nanostring between treatment naïve (TN) and previously treated (PT) patients. **B**; Flow cytometric analysis of inhibitory coreceptors and **C;** immunoblot analysis of phosphatases in three samples from untreated (UT) patients and three samples from previously treated (PT) patients. In **B**, graph shows results for individual samples and mean (±error). In **C**, GAPDH was analyzed as an additional loading control.

**Supplementary Figure 4: A**; Relative changes to surface IgM (sIgM) (GeoMFI) expression for 4 IcICLLe clinical trial patients between baseline and 1-month after initial receipt of ibrutinib treatment. **B**; Representative immunoblotting confirming presence of BTK within the input lysate of the baseline (BL) and 1-month (1M) treatment samples from a patient recruited to the IcICLLe clinical trial. **C**; Immunoblotting to illustrate loss of BTK binding to kinobeads through *in vivo* action of ibrutinib. Kinobead isolation was performed on matched samples for 2 representative patients, comparing the baseline sample to that taken 1-month following commencement of ibrutinib treatment. Kinobead elutions were mixed with loading buffer and separated by SDS-PAGE prior to blotting and probing.

**Supplementary Dataset 1:** Identities and average ratios gained for the kinome signature isolated using kinobead-MS experiments involving MEC-1 cells (n=3), comparing the impact of ibrutinib and dasatanib pre-treatment on kinase isolation by Ki-NET kinobeads.

**Supplementary Dataset 2:** Identities and intensities for kinase mRNA expression determined from Nanostring experiments comparing primary cells from treatment-naïve and previously treated CLL patients.

**Supplementary Dataset 3:** Identities and ratios gained for the kinome signature isolated using kinobead-MS experiments involving primary cells from CLL patients (n=40).

**Supplementary Table 1:** Characteristics of primary CLL samples analyzed from the main 40 patient cohort.
