# Supplementary figures and images for: Kinobead Profiling Reveals Reprogramming of BCR Signaling in Response to Therapy within Primary CLL Cells
Source: Clin Cancer Res. 2021 Aug 11;27(20):5647–59. doi: 10.1158/1078-0432.CCR-21-0161 (PMC9662893; doi:10.1158/1078-0432.CCR-21-0161)

Supplementary Figure 1

A

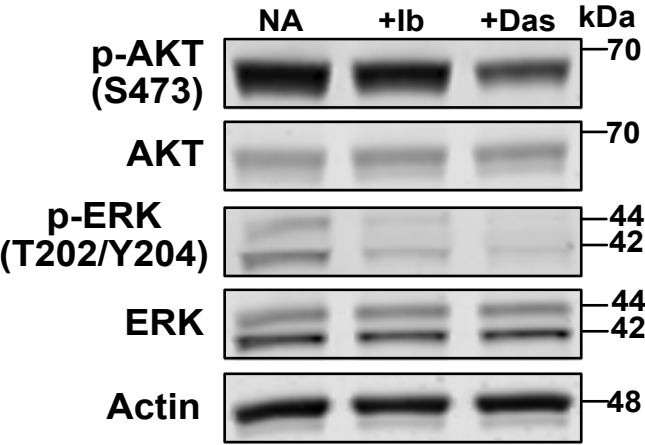

B

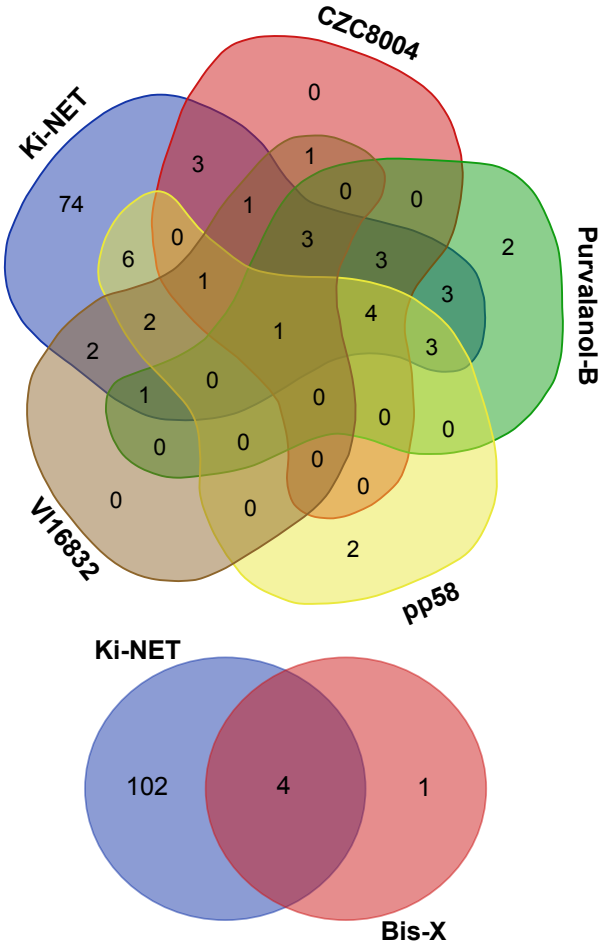

C

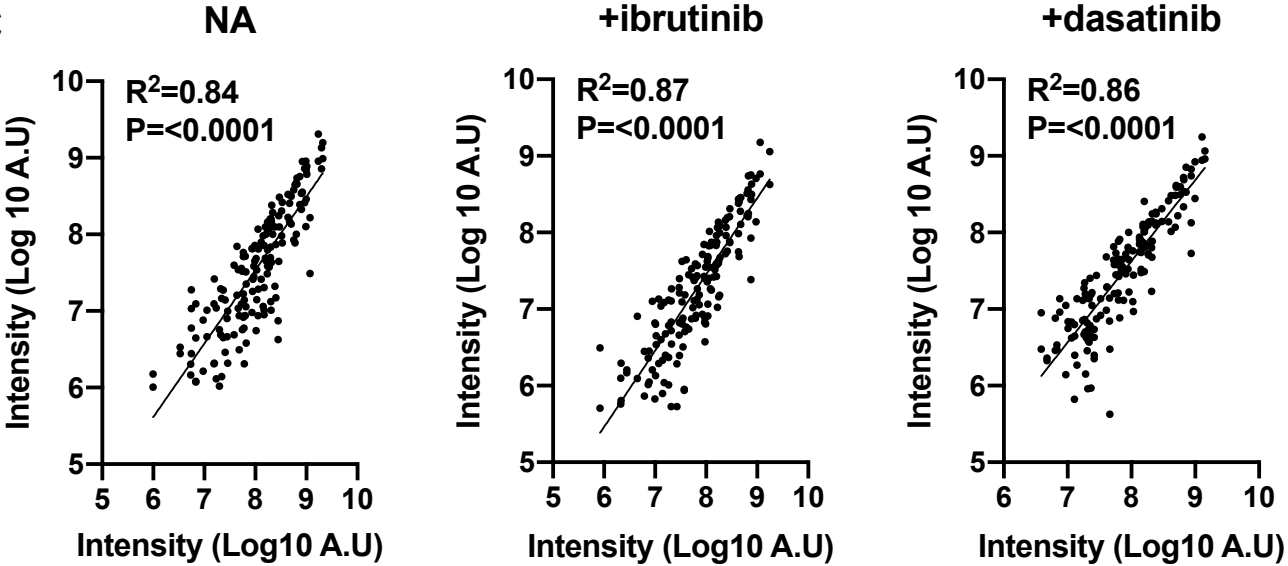

Supplement: Supplementary Figure 1 — A; Immunoblot analysis of MEC-1 cells showing expression of total and phospho-ERK1/2 and AKT in untreated control (NA), ibrutinib or dasatinib-pre-treated cells (both at 500 nM for 60 minutes). Actin was analyzed as an additional loading control. B; Venn diagrams showing overlap of kinases isolated by the different KI used to create kinobeads during this study. C; Correlation graphs comparing intensities (Log10 scale) for kinases isolated from MEC-1 lysates by Ki-NET beads in untreated, ibrutinib-and dasatinib pre-treated cells. [file 10780432ccr210161-sup-258762_3_supp_7227233_qvlxdk.pdf]

# Supplementary Figure 2

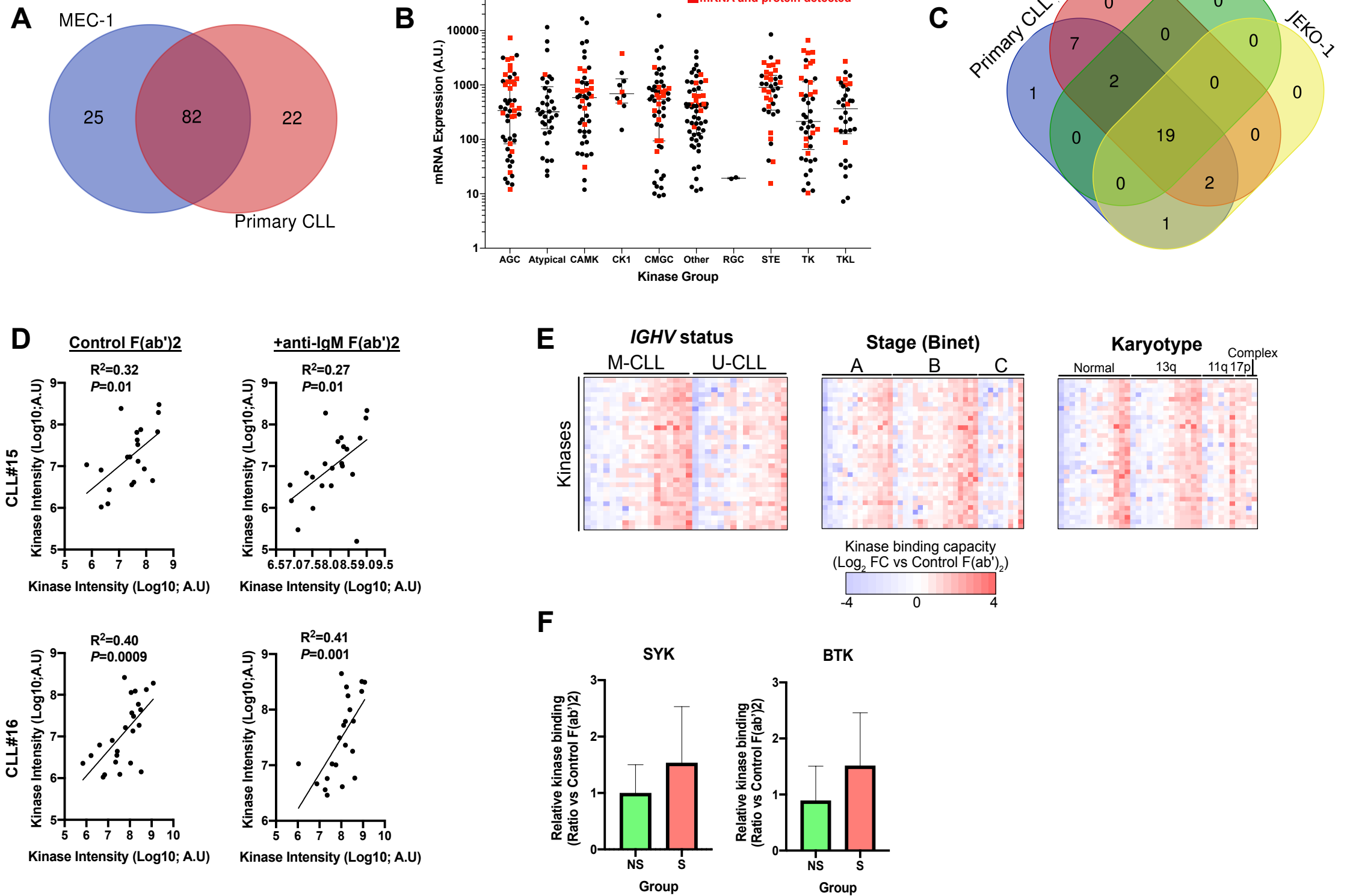

Supplement: Supplementary Figure 2 — A; Venn diagram showing overlap of larger kinobead signatures derived from MEC-1 and primary CLL cell experiments. B; Graph comparing kinases identified at either mRNA level, protein (by kinobead isolation) or both in our patient cohort. C; Venn diagram to compare overlap of the refined 32 kinase signature gained from primary CLL experiments in relation to the malignant lymphoid cell lines MEC-1, MAVER-1 and JeKo-1. D; Correlation graphs for 2 representative CLL patients, comparing intensities gained for our kinome signature for 2 biological repeats for baseline (Control F(ab')2) cells and in response to anti-IgM treatment. E; Heatmaps showing kinome fingerprints for CLL patients stratified according to IGHV mutation status, Binet staging or karyotype status. F; Graphs illustrating relative change in isolation of SYK and BTK in primary CLL cells from patients stratified according to in vitro iCa2+ flux response as being Non-Signaler (NS; iCa2<5%), or Signaler (S; iCa2+>5%). [file 10780432ccr210161-sup-258762_3_supp_7227234_qvlxdk.pdf]

# Supplementary Figure 3

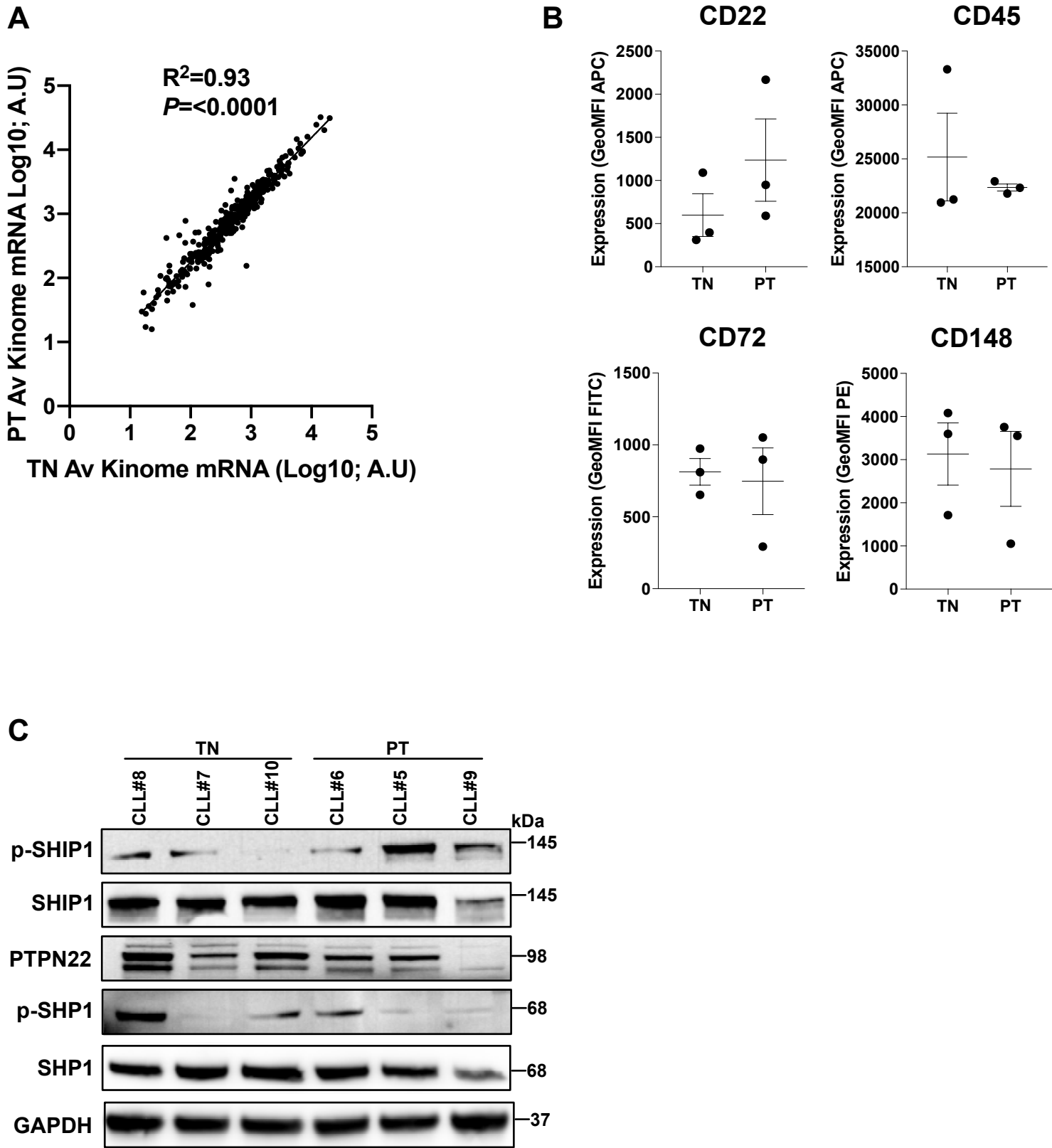

Supplement: Supplementary Figure 3 — A; Correlation of kinase mRNA expression determined by Nanostring between treatment naïve (TN) and previously treated (PT) patients. B; Flow cytometric analysis of inhibitory coreceptors and C; immunoblot analysis of phosphatases in three samples from untreated (UT) patients and three samples from previously treated (PT) patients. In B, graph shows results for individual samples and mean ({plus minus}error). In C, GAPDH was analyzed as an additional loading control. [file 10780432ccr210161-sup-258762_3_supp_7227235_qvlxdl.pdf]

Supplementary Figure 4

A

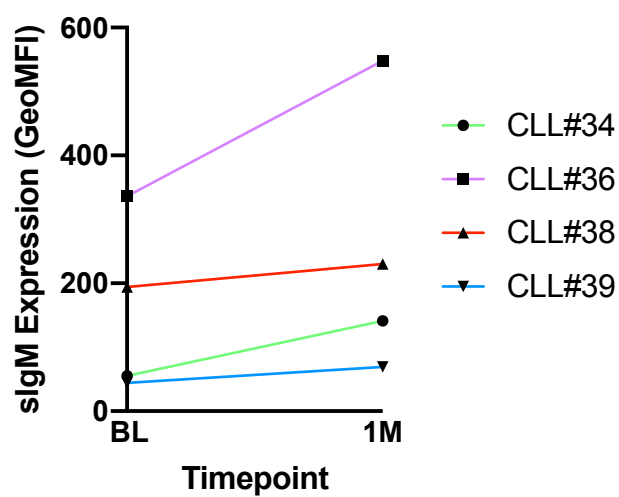

B

INPUT LYSATE

CLL#39

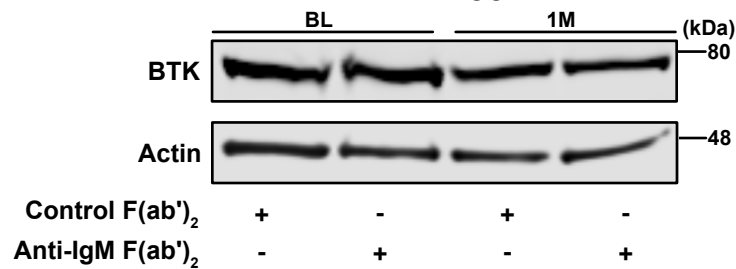

C

KINOBEAD ELUTION

CLL#46

CLL#47

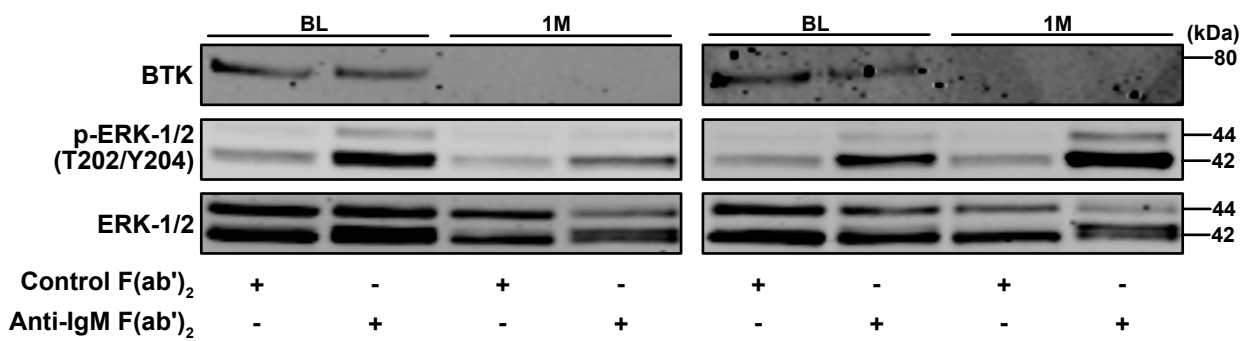

Supplement: Supplementary Figure 4 — A; Relative changes to surface IgM (sIgM) (GeoMFI) expression for 4 IcICLLe clinical trial patients between baseline and 1-month after initial receipt of ibrutinib treatment. B; Representative immunoblotting confirming presence of BTK within the input lysate of the baseline (BL) and 1-month (1M) treatment samples from a patient recruited to the IcICLLe clinical trial. C; Immunoblotting to illustrate loss of BTK binding to kinobeads through in vivo action of ibrutinib. Kinobead isolation was performed on matched samples for 2 representative patients, comparing the baseline sample to that taken 1-month following commencement of ibrutinib treatment. Kinobead elutions were mixed with loading buffer and separated by SDS-PAGE prior to blotting and probing. [file 10780432ccr210161-sup-258762_3_supp_7227236_qvlxdl.pdf]
